# Editorial: Exploring anthropometric markers in hormonal exposure and endocrine disease pathophysiology

**DOI:** 10.3389/fendo.2025.1600797

**Published:** 2025-04-17

**Authors:** Anna Kasielska-Trojan, Bogusław Antoszewski, John T. Manning

**Affiliations:** ^1^ Plastic, Reconstructive and Aesthetic Surgery Clinic, Institute of Surgery, Medical University of Lodz, Lodz, Poland; ^2^ School of Sport and Exercise Sciences, Applied Sports, Technology, Exercise, and Medicine (A-STEM), Swansea University, Swansea, United Kingdom

**Keywords:** anthropometric markers, digit ratio (2D:4D), endocrine diseases, BMI - body mass index, pathophysiology

Anthropometric measurements, encompassing body proportions and asymmetries, are pivotal in understanding prenatal development, postnatal growth, and the onset of various diseases. Anthropometric markers can also reveal early hormonal exposures. For instance, the digit ratio (2D:4D) is sexually dimorphic and serves as an indicator of 1^st^ trimester sex steroid exposure, with a lower ratio suggesting higher prenatal testosterone levels, as proposed by Manning et al. (1998) (1). Different 2D:4D patterns reflecting prenatal sex hormone exposure have been reported to be associated with a number of hormone-related diseases, e.g. idiopathic gynecomastia, autoimmune thyroid disorders, systemic lupus (2–4). Our Research Topic aimed to evaluate the role of wide spectrum of anthropometric markers indicating prenatal and postnatal hormone exposure, in understanding the pathophysiology of various diseases and conditions. We highlighted the investigation of the potential of anthropometric features, asymmetries, and ratios in identifying individuals at heightened risk of developing endocrine disorders. Additionally, we aimed to improve our understanding of how these markers can aid in diagnosing hormonal imbalances and endocrine diseases. Finally, we believe these insights can be used to inform public health strategies, such as targeted prophylactic examinations and educational campaigns for at-risk populations.

Life quality and its correlates are strongly associated with the concept of health and disease. Sexual functioning and the quality of sleep seem to be an important domain for the quality of life, with androgens playing a key role in its regulation for both men and women. In their series of two articles Bartoszek et al. and Bartoszek et al. aimed to examine these correlates of life quality from the aspect of prenatal androgens exposure (PAE) influence. Specifically, their goal was to explore potential correlations between the 2D:4D and sexual disorders, depression, anxiety, and sleep. With regard to sexual aspect, they found high PAE was associated with a greater openness to casual relationships, particularly among women, while low-PAE individuals prioritized intelligence over physical traits in partner preferences. These findings suggest that PAE, as measured by the 2D:4D ratio, may be associated with adult psychosocial traits. Innovatively, the authors explored the influence of PAE on sleep-related disturbances and found a link with sleep efficiency and chronotype. Men with lower PAE exhibited poorer sleep quality. Both studies highlighted the need for further research in more diverse populations and into gender-specific sleep regulation mechanisms.

Polycystic ovary syndrome (PCOS) is another common condition among women of reproductive age, with a prevalence around 10–15% (5). The syndrome is characterized by hormonal disorders, including hyperandrogenism, insulin resistance and thyroid dysfunction (6–9). Two articles addressed the problem of this condition regarding a predictive role of anthropometric variables and thyroid function in offspring birth features and adult cardiometabolic risk. Trouva et al. investigated whether maternal thyroid function influenced newborn anthropometrics in PCOS. They found that a higher maternal thyroxin in early pregnancy and its greater decrease during pregnancy was associated with a lower offspring birthweight and shorter birth length. It appears that subclinical variations in maternal thyroid function play a role in influencing birth anthropometrics of PCOS offspring. This further suggests that these anthropometric variables depend on hormones and can serve as markers for hormonal disorders. Valencia-Ortega et al. aimed to verify the relation between maternal concentrations of organokines (progranulin (PGRN), adipocyte fatty acid-binding protein (AFABP), brain-derived neurotrophic factor (BDNF), and fibroblast growth factor 21 (FGF21)) throughout pregnancy with anthropmetrics. The latter included neonatal weight and length at birth and at one month of age, in addition to the percentage of fat mass at one month of age. It appeared that these organokines and maternal characteristics can be useful in the prediction of neonatal weight, length, and percentage fat mass. However, Jabczyk et al. found that in women with PCOS variables used to assess body fat, such as BMI, BAI, WHR, and WHtR, appeared to be poor predictors of adult dyslipidemia.

Articles gathered in this Research Topic highlight the role of anthropometric features in understanding pathophysiology of some hormone-related disorders and abnormalities. These anthropometric traits may reflect hormonal concentrations during narrow developmental windows (e.g. 2D:4D as a marker for 1st trimester PAE) or throughout the prenatal period and into postnatal development (e.g. adiposity markers such as BMI, WHR). Such simple traits can aid in diagnosing hormonal imbalances and endocrine diseases. They may also give us information regarding prognosis, suggest treatment interventions and inform public health strategies.
